# Desensitization of Capsaicin-Sensitive Afferents Accelerates Early Tumor Growth *via* Increased Vascular Leakage in a Murine Model of Triple Negative Breast Cancer

**DOI:** 10.3389/fonc.2021.685297

**Published:** 2021-07-14

**Authors:** Noémi Bencze, Csaba Schvarcz, Gábor Kriszta, Lea Danics, Éva Szőke, Péter Balogh, Árpád Szállási, Péter Hamar, Zsuzsanna Helyes, Bálint Botz

**Affiliations:** ^1^ Department of Pharmacology and Pharmacotherapy, University of Pécs Medical School, Pécs, Hungary; ^2^ János Szentágothai Research Centre, Molecular Pharmacology Research Team and Centre for Neuroscience, University of Pécs, Pécs, Hungary; ^3^ Institute of Translational Medicine, Semmelweis University, Budapest, Hungary; ^4^ Department of Immunology and Biotechnology, University of Pécs Medical School, Pécs, Hungary; ^5^ 1st Department of Pathology and Experimental Cancer Research, Semmelweis University, Budapest, Hungary; ^6^ Institute for Translational Medicine, Medical School, University of Pécs, Pécs, Hungary; ^7^ Department of Medical Imaging, University of Pécs, Medical School, Pécs, Hungary

**Keywords:** breast cancer, *in vivo* imaging, 4T1 breast cancer, vascular leakage, capsaicin sensitive sensory nerves

## Abstract

There is growing interest in the role of nerve-driven mechanisms in tumorigenesis and tumor growth. Capsaicin-sensitive afferents have been previously shown to possess antitumoral and immune-regulatory properties, the mechanism of which is currently poorly understood. In this study, we have assessed the role of these terminals in the triple negative 4T1 orthotopic mouse model of breast cancer. The ultrapotent capsaicin-analogue resiniferatoxin (RTX) was used for the selective, systemic desensitization of capsaicin-sensitive afferents. Growth and viability of orthotopically implanted 4T1 tumors were measured by caliper, *in vivo* MRI, and bioluminescence imaging, while tumor vascularity and protease enzyme activity were assessed using fluorescent *in vivo* imaging. The levels of the neuropeptides Calcitonin Gene-Related Peptide (CGRP), Substance P (SP), and somatostatin were measured from tumor tissue homogenates using radioimmunoassay, while tumor structure and peritumoral inflammation were evaluated by conventional use of CD31, CD45 and CD3 immunohistology. RTX-pretreated mice demonstrated facilitated tumor growth in the early phase measured using a caliper, which was coupled with increased tumor vascular leakage demonstrated using fluorescent vascular imaging. The tumor size difference dissipated by day seven. The MRI tumor volume was similar, while the intratumoral protease enzyme activity measured by fluorescence imaging was also comparable in RTX-pretreated and non-pretreated animals. Tumor viability or immunohistopathological profile was measured using CD3, CD31, and CD45 stains and did not differ significantly from the non-pretreated control group. Intratumoral somatostatin, CGRP, and SP levels were similar in both groups. Our results underscore the beneficial, antitumoral properties of capsaicin sensitive nerve terminals in this aggressive model of breast cancer, which is presumed to be due to the inhibition of tumor vascular bed disruption. The absence of any difference in intratumoral neuropeptide levels indicates non-neural sources playing a substantial part in their expression.

## Introduction

Breast cancer is the most common malignancy, and also the most frequent cause of cancer-related death among women (1). Nearly 15% of all invasive breast cancers are triple-negative breast cancers (TNBC), a very aggressive form (2, 3) in which estrogen-, progesterone, and human epidermal growth factor receptor 2 (HER2) expression is absent, which necessitates finding novel targets to tackle TNBC. Since aggressive breast cancers are highly dependent upon vascular supply and neoangiogenesis, the 4T1 TNBC model (an aggressive mouse breast cancer cell line derived from the mammary gland of the BALB/c strain) is a vascularized tumor model (4). Due to poor vascularization of rapidly growing 4T1 tumors these develop spontaneous necrosis in the center of the tumor once reaching a certain size (5).

Neural factors bear profound influence on breast cancer growth and tumor cell proliferation, as summarized by a recent comprehensive review (6). Prior studies have shown that increased sympathetic activation facilitates metastatic potential in the 66cl4 murine mammary adenocarcinoma model (7). In the 4THMpc murine model, vagotomy also facilitated distant metastasis formation without influencing growth of the primary tumor, while pharmacological activation of the vagus nerve decreases metastatic potential coupled with an increased Substance P (SP)-level in sensory nerve endings (8, 9). While several preclinical studies demonstrated inhibitory vagal effects, multiple retrospective clinical studies regarding cancer patients indicated a stimulatory response. Thus, the net effect of parasympathetic activation remains controversial (10). More recently, neural activity and presence of nerve fibers have been confirmed *in vivo*, in orthotopic 4T1 breast tumors, and evoked responses were demonstrated when the cervical vagus nerve was electrically stimulated, indicating a direct neural connection between the brain and the mammary tumor. Intratumoral electrical activity may likely be inhibited through the use of chemical sympathectomy (11). Thus, neural regulation of the tumor microenvironment has become an area of immense interest, as recent evidence indicates tumor cells may commandeer and even reactivate otherwise dormant nerve-driven pathways to further facilitate their own growth (6).

Capsaicin-sensitive sensory nerves are selectively activated by capsaicin, the pungent alkaloid of hot peppers. Capsaicin activates the Transient Receptor Potential Vanilloid 1 (TRPV1) receptors, which is responsible for its distinct taste (12). If frequently activated, the receptors become desensitized towards further stimuli, which has already been exploited as a therapeutical approach found in nasal spray intended to treat migraine headaches, including the topical analgesic treatment of rheumatoid arthritis and sensory neuropathy (12). Recently, the clinical applicability of capsaicin has also emerged in oncology (13). Early on, the direct effect of capsaicin was debated (14), more recent studies overwhelmingly demonstrated anticancer effects, attributed to its pro-apoptotic, cell-cycle arrest-inducing, and anti-angiogenic properties (15–18). Capsaicin can ablate TRPV1-positive sensory neurons *via* Ca^2+^ overload. By a similar mechanism, it may also kill tumor cells which express TRPV1. Nonetheless, it remains to be seen, if tumors can indeed express TRPV1 at sufficiently high concentrations to effectively enable this killing effect. However, long-lasting effects of capsaicin and its analogues exerted *via* desensitization of capsaicin-sensitive afferents have manifold, indirect downstream effects. It has been demonstrated that capsaicin-induced desensitization of peptidergic sensory nerve terminals induces facilitated lung metastasis formation in the 4T1 breast cancer model. This was coupled with an increased number of metastatic deposits in the heart, an otherwise uncommon site for metastasis formation in the model. Interestingly, lower, non-desensitizing doses of capsaicin were found to diminish lung metastasis formation. Comprehensively, it has been postulated, increased activation of capsaicin-sensitive afferents bears protective effects, while their defunctionalization is detrimental, by removing the homeostatic effects exerted by the neuropeptides released from them (19). As afferents, capsaicin-sensitive peptidergic nerves transmit inflammatory signals from the tumor to the central nervous system. Furthermore, they also act as efferents, *via* neuropeptides released locally from peripheral capsaicin-sensitive nerves and may also influence tumor growth. Since many of these neuropeptides are potently vasoactive, such as SP, Calcitonin Gene-Related Peptide (CGRP), while others are anti-inflammatory (e.g., somatostatin), the net effect of their actions can be considerably diverse. The sensory nerves in the tumor microenvironment (stroma) are also known to regulate neoangiogenesis in the tumor, and may play an important role in tumor formation and progression. Capsaicinoids demonstrate robust anti-angiogenic activity in mouse models (20). Vagal afferentation was also found relevant in tumorigenesis and tumor growth. It has been shown by several prospective clinical studies, vagotomy increases the risk of developing lung or colorectal cancers later on, and *in vivo* studies have demonstrated increased metastatic potential in breast cancer following vagotomy. It has also been demonstrated the sensory component of the vagal nerve also contains capsaicin-sensitive afferents (8, 9, 21).

In this study, we aimed to characterize the role of capsaicin-sensitive fibers in a mouse model of an orthotopically implanted, triple negative 4T1 breast cancer model, using functional assessment of tumor growth, *in vivo* imaging of tumor structure, vascularity, and inflammatory enzyme activity. This was followed by *ex vivo* evaluation of intratumoral levels of key neuropeptides contributing to their vaso-, and immunoregulatory actions, including conventional and immune-histopathological assessment of the tumor stroma. This is an important question from a clinical perspective too, since high-dose capsaicin patches which function by sensory desensitization are already in clinical use to relieve neuropathic pain (22). Thus, in this study we aimed to determine breast cancer progression in mice after defunctionalizing the capsaicin-sensitive peptidergic sensory nerves by pretreatment with the ultrapotent capsaicin analog resiniferatoxin (RTX), and to identify the factors which may influence tumor growth.

## Materials and Methods

### Animals

Six-to-eight week old female BALB/c mice were bred and kept at the Laboratory Animal House of the Department of Pharmacology and Pharmacotherapy of the University of Pécs at 24-25°C, and provided with standard rodent chow and water ad libitum under 12h dark/12h light cycles. The study was designed and conducted in full accordance to the European legislation (directive 2010/63/EU) and the Hungarian Government regulation (40/2013., II. 14.), in reference to the protection of animals used for scientific purposes. The project was approved by the Animal Welfare Committee of the University of Pécs, and the National Scientific Ethical Committee on Animal Experimentation of Hungary and licensed by the Government Office of Baranya County (license No. BA02/2000-32/2018). All efforts were made to keep the number of experimental animals involved in the study at the necessary minimum.

### Sensory Desensitization by RTX Pretreatment

The involvement of capsaicin-sensitive neurons in tumor progression was investigated with their long-lasting desensitization using systemic treatment with high doses (10, 20, 70 and 100 µg/kg) of the ultrapotent capsaicin analogue RTX (Sigma Aldrich, St. Louis, MO, USA) injected subcutaneously over the span of four consecutive days) three weeks prior to the inoculation (23). Mice were simultaneously treated with a solution containing 4% terbutaline–sulfate (AstraZeneca Ltd., Hungary, 4% theophylline-ethylene diamin (Gedeon Richter Plc., Hungary) and 2% atropine–sulfate (Egis Pharmaceuticals Plc., Hungary). The supporting drug cocktail was utilized to diminish the acute respiratory side effects of RTX-pretreatment (primarily bronchoconstriction and increased mucus secretion). The supporting mixture has been shown to reduce animal loss during desensitization with high dose capsaicin and its analogs, including RTX (24, 25). RTX elicits permanent opening of the TRPV1 ion channel receptors, and the resultant intracellular cation influx induces long-lasting defunctionalization of the whole sensory nerve terminals. Thus, it acts as a selective neurotoxin, through which all TRPV1 receptor-expressing afferents are deactivated. RTX has been shown to be several magnitudes (100 to 200 fold) more potent TRPV1 receptor agonist compared to capsaicin. It was also proven that the loss of TRPV1 receptor expression in the central nervous system is not reversible following RTX-pretreatment (26, 27). As RTX-pretreated mice do not display nocifensive reactions against TRPV1 agonist irritants (e.g., capsaicin), these animals are colloquially referred to as desensitized. The success of the sensory defunctionalization was confirmed two weeks later by the absence of increased eye-wiping behavior following 10 µl, 0.1% capsaicin drop compared to its vehicle, indicating the peripheral defunctionalization of peptidergic afferents. This test is a routinely used, well-established and widely accepted technique (24, 28). In a prior study we have reported a striking difference in the number of eye wipings in RTX-pretreated mice (less than five per minute) compared to non-pretreated mice (over 30 per minute) (23). As the 0.1% capsaicin solution elicits a very profound nocifensive response in intact mice, in this study it was only administered to RTX-pretreated animals.

### Tumor Model

4T1 tumor cells (29) transfected with firefly luciferase were grown in cell culture and processed for inoculation *in vivo* (5). The cell line was maintained in DMEM (Gibco, Invitrogen) with 10% FBS, 1 mM l-glutamine, and penicillin/streptomycin (Gibco; Invitrogen) and 0.25% trypsin/1mM EDTA (Life Technologies). Animals were anesthetized by 100 mg Ketamine/10 mg Xylazine/kg (Calypsol^®^ 50 mg/ml, Richter Gedeon, Budapest, Hungary; CP-Xylazin 2%, Produlab Pharma, Raamsdonksveer, Netherlands) intra-peritoneally (i.p.) 1x 10^6^ 4T1 cells in 50 µl 1:1 Matrigel^®^-PBS (Matrigel^®^ Basement Membrane Matrix, 354234, Corning^®^, NY, USA; Phosphate Buffered Saline without Calcium and Magnesium #17-516F, Lonza A. G., Basel, Switzerland) solution were subcutaneously inoculated by a 50 μl Hamilton syringe (Hamilton Company, Reno, Nevada, US). Inoculation was made orthotopically into the fourth mammary gland’s fat pad in each mouse (30). Animals with the largest, smallest or twin tumors were excluded from the study.

### Tumor Size Measurements

Tumor size was measured daily using a digital caliper. Mice were held in hand while measuring all three dimensions (π/6 x length, x width and x height) of each tumor using the digital caliper with 0.01 mm precision (Mitutoyo, Kawasaki, Japan). The length was measured along the longest linear dimension on the skin and the width along the axis perpendicular to the longitudinal axis. The height was measured at the tallest point of the tumor (31).

### *In Vivo* Bioluminescence Imaging of Tumor Growth and Viability

On days one, three and eight, following the inoculation of bioluminescent tumor cells, the animals underwent *in vivo* imaging using the IVIS Lumina III imaging system (PerkinElmer, Waltham, MA, USA). The transfected 4T1 tumor cells express firefly luciferase which produces bioluminescence following the addition of its substrate (luciferin). In consideration of the imaging, a 15 mg/ml solution of D−luciferin sodium salt (Gold Biotechnology, St. Louis, MO, USA) in DPBS was administered i.p. in a 150 mg/kg dose. Animals were anesthetized using ketamine-xylazine three minutes following the administration of D-luciferin, and were imaged ten minutes postinjection using the following settings: auto acquisition, F/stop = 1 and Binning = 4. Data were analyzed using the Living Image software (PerkinElmer). Regions of interest (ROI) were drawn around the luminescent tumor signal automatically using identical signal thresholds. Total radiance, a calibrated unit of the luminescence (total photon flux per second), was calculated in each ROI and used for further statistical analysis.

### *In Vivo* Fluorescence Imaging of Protease Activity and Tumor Vascular Leakage

Increased levels of protease enzyme activity is a hallmark feature of many tumors including breast cancer. Protease enzyme activity was measured *in vivo* with the ProSense 680 (PerkinElmer) activable fluorescent probe using the FMT 2000 fluorescence molecular tomography system (PerkinElmer). ProSense is selectively activated by several important, disease-associated proteases, such as Cathepsin B, L, S and Plasmin, and was previously shown to be a robust tool in monitoring their activity. ProSense is a fluorophore-conjugated graft copolymer of polyethylene glycol and poly(Lys), and it is optically silent under normal conditions. Upon selective enzymatic cleavage the probe regains its fluorescent properties in the near-infrared range, thus allowing selective detection of enzymatic activity (32). ProSense was reconstituted in sterile 1xPBS. The probe was injected intravenously (IV) under anesthesia through the retroorbital venous plexus in a dose of two nmol/subject fully compliant to the manufacturer’s recommended protocol. The mice were consequently gently shaved and topically treated with hair removal cream under anesthesia in the area surrounding their torso, in order to reduce optical signal loss and scatter. Three-dimensional tomographic imaging was performed twenty-four hours postinjection using the 680 nm laser channel of the device. In the 3-dimensional volumes of interest representing the tumor tissue, the amount of fluorophore was automatically calculated through the use of TrueQuant software (PerkinElmer) of the instrument in picomoles.

Tumor vascular leakiness was assessed with the AngioSense 680EX (PerkiElmer) fluorescent blood pool imaging probe in a different set of mice. Upon IV injection, AngioSense remains localized in the vasculature under normal conditions for up to four hours, only showing accumulation in regions where vascular structure is distorted, either due to pathological vessel leakage or neoangiogenesis. The probe was injected (two nmol/subject in 1xPBS) and mice were prepared for imaging as formerly described. Imaging was performed at twenty-four hours postinjection to eliminate the biasing effect of any circulating tracer. The amount of fluorophore in the tumor tissue was quantified as described earlier.

### Magnetic Resonance Imaging of Tumor Size and Internal Structure

The time course regarding tumor development was followed up with an MRI from each treatment group on the second and on the sixth days using a Bruker PharmaScan^®^ (4.7 T) small-animal MRI instrument. The imaging protocol was performed with a fast T2-RARE (Rapid Acquisition with Relaxation Enhancement) method (Thk: 1.00 mm, TR: 4000.00 ms, TE: 40.00 ms, matrix: 256x256). Anesthesia was induced by 3% V/V of isoflurane (Medicus Partner) in a gas mixture of 33% O_2_ and 66% N_2_O *via* rodent facemask and maintained with 1–2% isoflurane controlled by respiratory monitoring and gating system. Data were analyzed using the 3D-Slicer open access software (33, 34).

### Radioimmunoassay Measurement of Neuropeptide Concentration in the 4T1 Tumor Tissue

CGRP, SP and somatostatin concentrations were determined from homogenates of the 4T1 tumors. RIA was performed in phosphate buffer (pH 7.4, 50 mmol/L) containing sodium chloride (100 mmol/L), sodium azide (0.05% w/v) and bovine serum albumin (0.25% w/v). Polypropylene assay tubes contained samples or standard peptides, tracer, antiserum and assay buffer. Standard, tracer and antiserum were dissolved in an assay buffer. Samples measured 500 µL, whereas standard, tracer and antiserum were 100 µL each. Tubes containing standard received 500 µL SIF solution to mimic the effect of the sample solvent. Tubes were filled to 1000 µL with assay buffer. Somatostatin-14, Tyr-α-CGRP23–37 and Lys3 Substance P (Sigma-Aldrich, Hungary; Bachem, USA; and PerkinElmer, USA, respectively) were used as a standard. The tracer was produced from (Tyr1)-somatostatin-14 and Tyr-α-CGRP23–37 by incubating the peptide with Na-^125^I and 1, 3, 4 and 6-tetrachloro-3a and 6a-diphenyl-glycoluril. The mono-iodinated peptide was separated during the reverse phase HPLC column. Tracer dilution was adjusted to allocate 3000 cpm/100 µL. In the case of SP, ^125^I-Bolton Hunter labeled Lys3 Substance P was used. Antiserum was used at 1:250000, 1:190000 and 1:320000 dilution in the case of SOM, CGRP and SP, respectively. Assay tubes were incubated at 4° C for 48-72 h. Antigen-bound and free peptides were separated by adding 100 µL of a disperse system containing Norit-A (10% w/v), dextran (MW: 65-73 kDa, 1% w/v) and commercial fat-free milk powder (0.2% w/v) in distilled water. Tubes were centrifuged at 4° C, 3000 g, for twenty minutes. Pellets containing free peptides and supernatant containing antibody-bound peptides were separated and radioactivity of both was detected (Gamma NZ-310, Hungary). A standard curve was generated by plotting the ratio of the activity of pellets and the activity of supernatants of standard samples. Results are expressed as total SOM-like, CGRP-like and SP-like immunoreactivity (fmol SOM-LI, fmol GGRP-LI and fmol SP-LI) per mg tissue weight or per ml.

### Histopathology and Immunohistochemistry

Procedures were performed as formerly described (35). Briefly, tumor tissue samples were fixed in 10% formalin, dehydrated and embedded in paraffin, followed up with serial sections (2.5 µm) which were cut. Sections were dewaxed and rehydrated for hematoxylin-eosin (H&E) staining and immunohistochemistry. In consideration of antigen retrieval, heating was applied for twenty minutes in Tris-EDTA (TE) buffer pH 9.0 (0.1 M Tris base and 0.01 M EDTA) using an Avair electric pressure cooker (ELLA 6 LUX(D6K2A), Bitalon Ltd., Pécs, Hungary). and twenty minutes cooling with open lid. Endogenous peroxidase blocking was performed using 3% H_2_O_2_ in methanol for fifteen minutes. Non-specific proteins were blocked for fifteen minutes in 3% bovine serum albumin (BSA, #82-100-6, Millipore, Kankakee, Illinois, USA) diluted in 0.1 M Tris- buffered saline (TBS, pH 7.4) containing 0.01% sodium azide. Sections were incubated with primary antibodies diluted in 1% BSA/TBS + TWEEN (TBST, pH 7.4) overnight (CD31), for sixty (CD45), and ninety minutes (CD3) in a humidity chamber. Peroxidase-conjugated anti-rabbit and anti-mouse IgGs (HISTOLS-MR-T, micropolymer -30011.500T, Histopathology Ltd., Pécs, Hungary) were used for forty minute incubations and the enzyme activity was revealed with three each, 3’-diaminobenzidine (DAB) chromogen/hydrogen peroxide kit (DAB Quanto-TA-060-QHDX-Thermo Fischer Scientific, Waltham, MA, USA) under microscopic control. All incubations were set to room temperature with the samples washed between incubations in TBST buffer for 2 x 5 min. H&E, CD3 (FLEX Polyclonal Rabbit Anti-Human CD3 RTU, produced by DAKO), CD31 (clone D8V9E, produced by Cell Signaling Technology) and CD45-immunostained (clone M0701, produced by DAKO) slides were digitalized by Pannoramic Digital Slide Scanner (3DHISTECH Ltd., Budapest, Hungary). Digitalized slides were evaluated in CaseViewer image-analysis software (3DHISTECH, Ltd.) with the QuantCenter-HistoQuant module. Specific CD3, CD31 and the CD45 signal was masked according to chromogene reaction intensity on the sections in the annotated area (entire tumor or inflammatory ring area). The ratio of masked and annotated area (relative mask area, rMA (%)) was used to estimate CD3, CD31 and CD45 expression. CD31 expression was evaluated in regards to the entire tumor annotated area. CD3 and CD45 expression was evaluated in the annotated area of the inflammatory ring regarding CD3 and CD45 stained slides, respectively. Inflammatory ring/tumor area ratio was calculated on CD3 stained slides.

### Statistical Analysis

**Figure 1:**
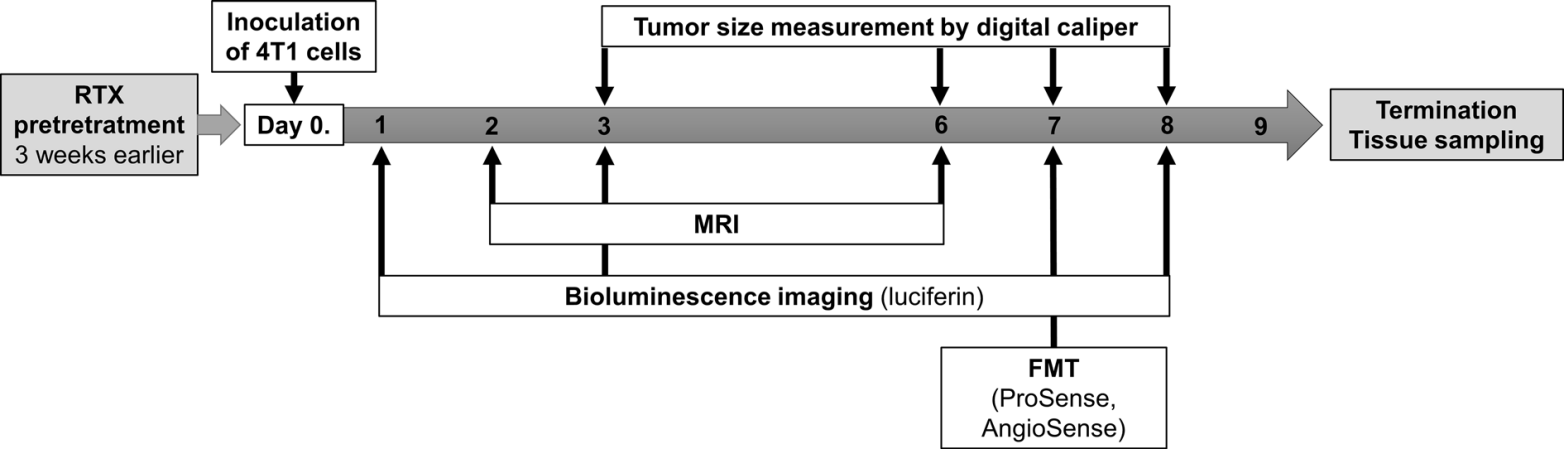
Summary of the experimental design and timeline including the timing of the resiniferatoxin (RTX) pretreatment, inoculation of tumor cells (4T1), tumor size measurements by caliper, MRI, as well as *in vivo* luminescence and fluorescence imaging by IVIS and FMT.

All data are presented as means ± SEM, and were analyzed using the statistical software package, GraphPad Prism v.7. (GraphPad Software, Inc., San Diego, CA, USA). Results were analyzed by two-way analysis of variance (ANOVA) followed by Sidak’s multiple comparison test. Fluorescence *in vivo* imaging (AngioSense, ProSense), RIA (CGRP, SP, and somatostatin) and immunohistopathology data were analyzed first by unpaired t-test and F-test. Due to the significantly different variance of the groups in two datasets (AngioSense, SP-levels), these were further analyzed by the modified Welch t-test. The experimental layout has been summarized in **Figure 1**.

## Results

### Increased Tumor Burden in the Early Phase of Tumor Growth in Desensitized Mice

**Figure 2:**
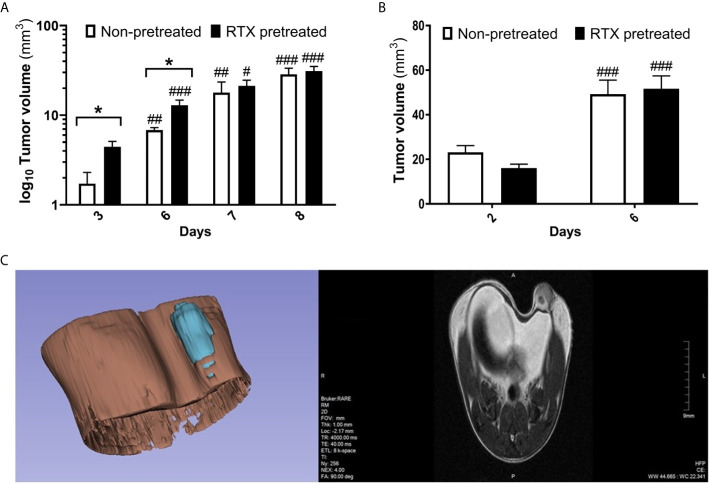
Moderately increased tumor volume in RTX pretreated mice in the early phase postimplantation. **(A)** Primary 4T1 tumor volume (mm^3^) and growth in BALB/c mice (n = 10-14 per group, log_10_ scale), measured using a digital caliper. **(B)** Tumor size measurement (mm^3^) evaluated by high resolution MRI. (n = 10). **(C)** Representative image of tumor structure (3D volume rendering and one T2-weighted slice in the coronal plane). Tumor volumes were calculated *via* 3D rendering and measuring. Data were calculated with 3D-Slicer 4.10.2, *p < 0.05 *vs*. non-pretreated, ^#^p < 0.05, ^##^p < 0.01, ^###^p < 0.001 *vs*. initial measurement of the respective group.

Following the orthotopic injection of the 4T1 tumor cells, rapid growth was observed, with the mean tumor volume reaching 28.58 and 31.03 mm^3^ by day eight in non-pretreated and RTX-pretreated animals, respectively. In the early phase of tumor growth, particularly on day three and six, larger tumor volumes were found using a digital caliper in desensitized mice when compared with those in controls (4.55 *vs*. 1.72 and 21.25 *vs*. 17.87 mm^3^, p=0.0204 and 0.0375 respectively, **Figure 2A**), and to a lesser degree, also on day seven and eight.

Direct volumetric T2-RARE MR imaging also demonstrated rapid tumor growth, with the mean tumor volume increasing from 23 and 16 mm^3^ to 49 and 51 mm^3^ in non-pretreated and desensitized mice respectively between day two and six. However, no significant difference in tumor volume could be observed between the groups on MRI (**Figures 2B, C**).

### 4T1 Tumor Cell Viability and Growth Measured by Bioluminescence Is Not Significantly Different in Desensitized Mice

**Figure 3:**
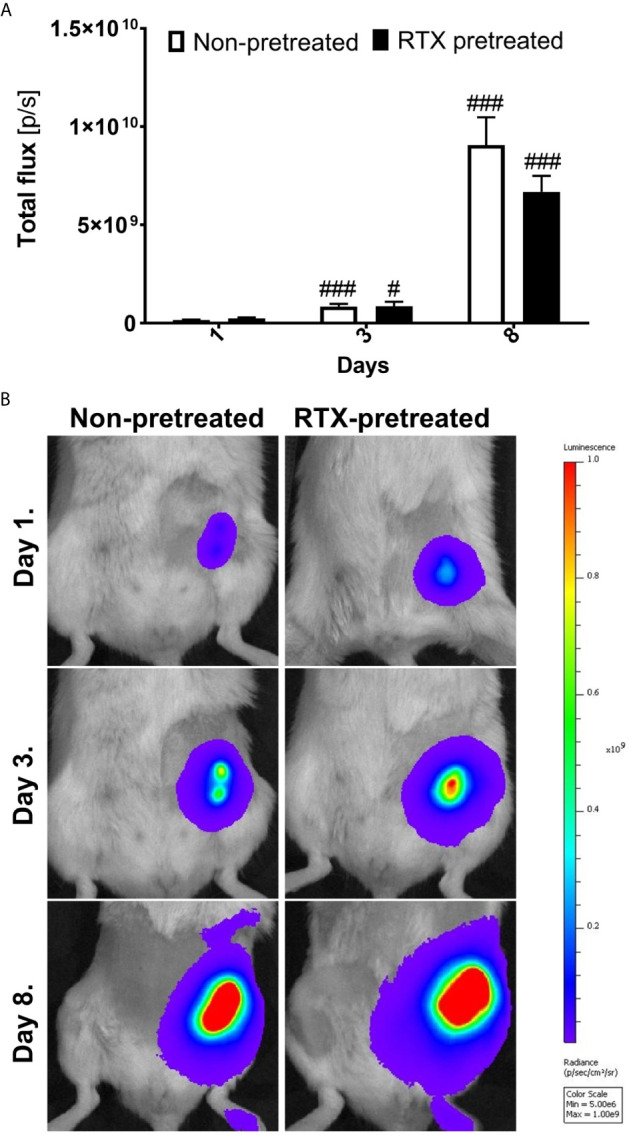
Similar 4T1 tumor cell viability and growth measured by bioluminescence imaging in desensitized mice. **(A)** Tumor viability measured as the luminescence (total flux [p/s]) of luciferase transfected 4T1 tumor cells non-pretreated and RTX pretreated groups (n=10-14/group). **(B)** Representative *in vivo* bioluminescence images shown as pseudocolor representation of radiance [p/sec/cm^2^/sr] overlaid onto simultaneously acquired grayscale photographs of the subjects. The color scale indicates the level of luminescence corresponding to each color on the images, ^#^p < 0.05, ^###^p < 0.001 *vs*. initial measurement of the respective group.

Immediately twenty-four hours following the inoculation of the 4T1 cells, the *in vivo* luciferase bioimaging clearly demonstrated the viability of cancer cells, confirming the success of the orthotopic implantation. By day three, the bioluminescence signal strength reflecting the viable cancer cells increased significantly to nearly three to fourfold, which was followed by an additional increase, approaching tenfold, in luciferase luminescence by day eight. However, no significant difference could be observed between the desensitized and control groups (**Figures 3A, B**).

### Similar Protease Activity, Yet Increased Tumor Vascular Leakage Measured Using Fluorescence *In Vivo* Imaging in RTX-Pretreated Animals

**Figure 4:**
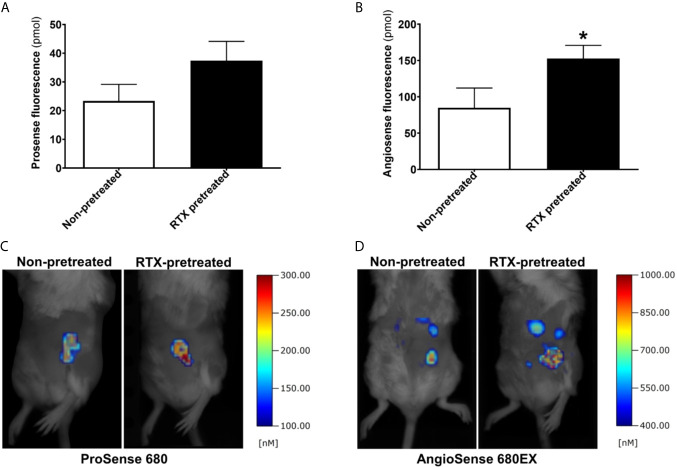
Increased intratumoral vascular leakage in RTX pretreated animals. **(A, B)** Fluorescent molecular tomography (FMT) imaging of protease enzyme activity (ProSense 680) and tumor vascular leakage (AngioSense 680EX) (n = 5-7/group) measured as the amount of fluorescent tracer in the tumors (pmol) on day seven following inoculation. **(C, D)** Representative FMT-images shown as pseudocolor representations of the amount of fluorophore, overlaid onto grayscale silhouette images of the subjects, *p < 0.05 *vs*. non-pretreated.

Protease activity within the 4T1 tumors was measured by the enzyme-activatable fluorescence probe ProSense on day seven following inoculation. 4T1 tumors demonstrated robust and specific enzyme activity at this point in both RTX-pretreated and control animals. The protease activity tended to be greater in desensitized mice, however, the difference was not significant (**Figures 4A, C**).

Tumor vascularity and leakage were also measured using the AngioSense fluorescent tracer on day seven. Since the measurement was carried out 24 hours postinjection, the fluorescence signal observed at this time point is representative regarding the retained tracer due to the enhanced permeability and retention effect characteristic of malignant tumors including breast cancer. The vascular signal was significantly increased in desensitized mice compared with their non-pretreated controls (mean tracer amount in tumors 154.8 *vs*. 84.9 pmols, p=0.039) indicating increased tumor vessel leakage (**Figures 4B, D**).

### No Difference in the Intratumoral Concentration of the Neuropeptides CGRP, Somatostatin and SP in Desensitized Mice

**Figure 5:**
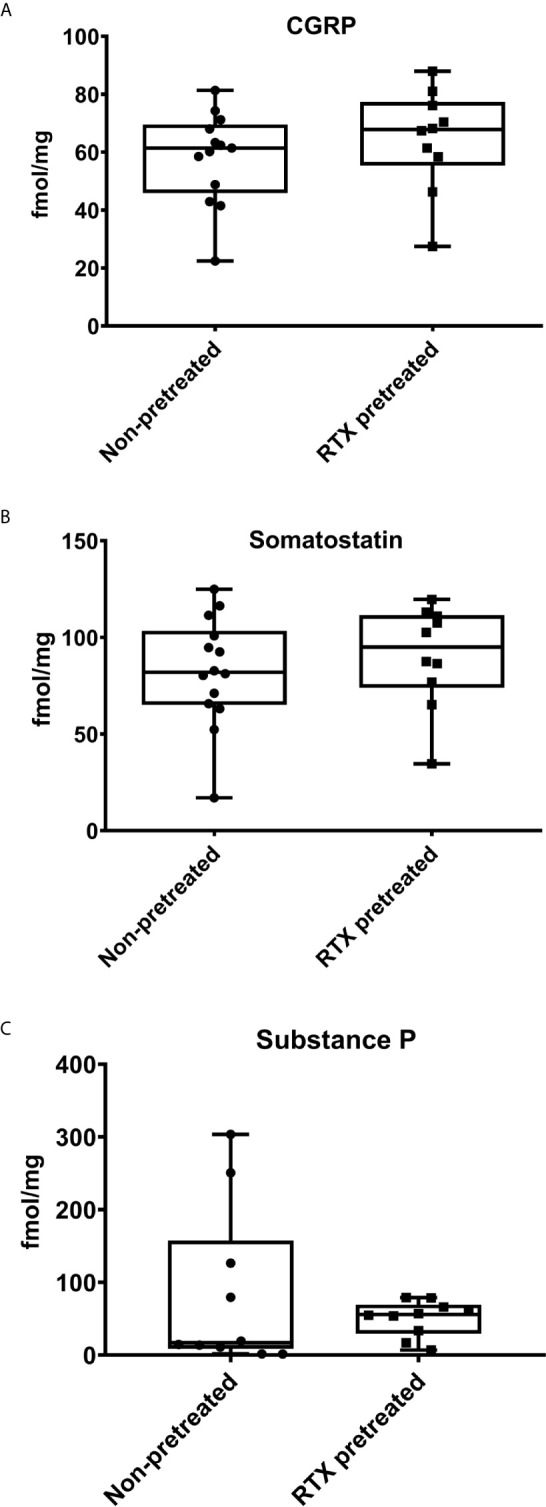
Intratumoral concentrations of the neuropeptides Calcitonin Gene-Related Peptide (CGRP), Somatostatin, and Substance P (SP) measured by radioimmunoassay are not significantly altered by RTX-pretreatment. Intratumoral neuropeptide concentrations (fmol/mg) of **(A)** CGRP, **(B)** Somatostatin, **(C)** SP. The min to max values are demonstrated with all data points.

Neuropeptides measured by RIA from tumor tissue homogenates obtained at the end of the experiment revealed no significant differences in the concentration of the proinflammatory and vasoactive CGRP, and the anti-inflammatory somatostatin between desensitized mice and the non-pretreated control group (**Figures 5A, B**). The concentration of the predominantly proinflammatory, pronociceptive, and vasoactive neuropeptide SP was also not significantly lower in RTX-pretreated mice compared to their controls (50.9 *vs*. 82.2 fmol/mg tumor tissue; **Figure 5C**).

### Unaltered Levels of CD31, CD3, and CD45 Immunoreactivity in the 4T1 Breast Tumor Tissue in Desensitized Mice

**Figure 6:**
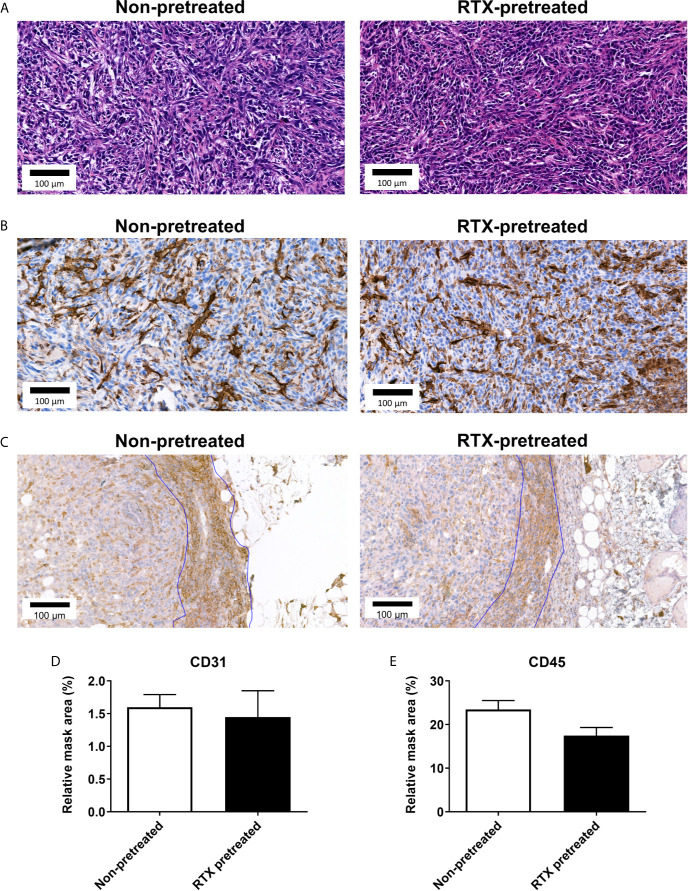
No significant difference observed in in CD31 and CD45 immunoreactivity of the 4T1 tumor tissue in RTX-pretreated mice. **(A)** Representative H&E and **(B)** anti-CD31 stained histopathological slides with high magnification (23x). **(C)** Representative anti-CD45 stained slides with high (23x) magnification. **(D)** CD31 expression measured by quantitative immunohistochemistry (relative mask area %). **(E)** CD45 expression measured by quantitative immunohistochemistry (relative mask area %).

Considering the observed *in vivo* differences in intratumoral vascular leakage, CD3, CD31, and CD45 were selected as markers to further assess endothelial changes and immune-cell response (36–38). Semi-automated quantification of CD31 immunohistology within the annotated area of the tumor tissue harvested at the end of the experiment was not significantly different in RTX-pretreated and non-pretreated control animals (1.449 *vs* 1.599 relative mask area (rMA) (%); **Figures 6A, B, D**). An inflammatory ring in the area adjacent to the tumors appeared in both the non-pretreated and RTX-pretreated group following H&E staining.

The vast majority of inflammatory cells in the rings appeared to be small cells with round nuclei, identified as T-lymphocytes (not shown). CD45 expression analysis also showed no difference in the inflammatory ring area (23.48 *vs*. 17.50 rMA(%); **Figures 6C, E**).

**Figure 7:**
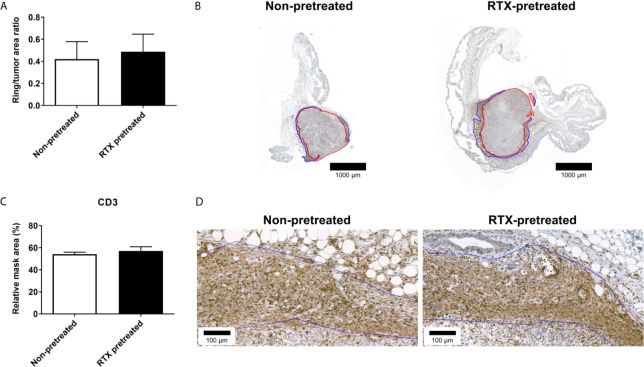
No significant difference in the CD3 immunoreactivity-based inflammatory response in the area surrounding the 4T1 tumors after RTX-pretreatment. **(A)** Ratio of the inflammatory ring area to the entire tumor area **(B)** Representative anti-CD3 stained sections, demonstrating tumor annotation (red) and the inflammatory ring (blue) with low (1.4x) magnification. **(C)** CD3 expression measured by quantitative immunohistochemistry (relative mask area %). **(D)** Representative anti-CD3 stained slides with high (23x) magnification. The inflammatory ring is marked with blue annotation.

A more detailed analysis performed through anti-CD3 immunohistochemistry however, revealed no difference between RTX-pretreated and non-pretreated tumors: The extent of the inflammatory rings (inflammatory ring area/tumor area: 0.49 *vs*. 0.42 ring/tumor area; **Figures 7A, B**) and quantification of CD3+ (57.04 *vs*. 54.60 rMA(%); **Figures 7C, D**) in the inflammatory rings were similar.

## Discussion

In consideration of this model of 4T1 orthotopic, triple negative breast cancer, we found divergent effects of RTX-induced desensitization of the capsaicin sensitive afferents. First, we demonstrated in the early period following tumor tissue implantation, growth was accelerated in RTX-pretreated mice; however, this difference subsides in the later phase. The facilitated tumor growth is accompanied by increased intratumoral vascular leakage, while inflammatory enzyme activity does not significantly differ. Notably, tumor expansion measured through the use of bioluminescence imaging is also comparable in desensitized and control mice. CGRP, somatostatin, and SP levels measured in the tumor tissue showed no significant difference between the groups. Histological tumor tissue morphology, including CD31, CD3 and CD45 expression measured by immunohistochemistry, did not differ significantly between the groups, indicating tumor architecture itself is not directly influenced by RTX-pretreatment. The interesting discrepancy between the tumor size measured through the use of a caliper and MRI can be attributed to the fact the latter method quantified tumor volume, yet not the peritumoral edema, which is often significant in aggressive forms of breast cancer (39).

The increased early growth and vascular leakage in the orthotopically implanted 4T1 tumors of desensitized mice indicates a beneficial regulatory effect of capsaicin-sensitive sensory nerves. Many neuropeptides released from these terminals (including CGRP and SP) are amongst the most potent endogenous vasoactive mediators, and can induce vasodilation, increased vascular leakage and increased efflux of plasma proteins and leukocytes (40–42). From a practical viewpoint, the observed increased intratumoral leakage following desensitization is a Janus-faced feature. Hyperpermeability of tumor vessels can hinder effective drug delivery, yet can also facilitate leakage and accumulation of large molecule drugs which cannot readily leave normal vasculature, resulting in their selective accumulation in the tumor tissue (43). Our results are in agreement to a prior study in which pretreatment with the less potent TRPV1-agonist capsaicin facilitated the metastasis of 4T1 tumor in the lung and heart, while in lower, non-desensitizing doses, it decreased the frequency of lung metastases (19). In this experiment, growth of the primary implanted tumor was unaffected by capsaicin-induced desensitization. However, the authors did not evaluate tumor growth in the very early phase postimplantation.

Among the neuropeptides released by capsaicin-sensitive afferents, SP was shown to facilitate breast cancer growth and metastasis formation (44). In our model, SP levels did not change significantly, despite increased early phase tumor growth and vascular leakage. The unaltered CGRP, SP and somatostatin levels observed in the tumor tissue of desensitized mice indicate significant non-neural sources of these mediators. The absence of the beneficial immuno-regulatory effect of sensory neuropeptides released by sensory nerve endings had been previously suggested as a detrimental factor in cancer, since SP at low levels can increase immunoglobulin and cytokine production, facilitates B-cell differentiation and T-lymphocyte proliferation (19, 45–48). Furthermore, regarding metastatic 4T1 breast cancer, it was discovered that SP is capable of counteracting tumor-induced endothelial dysfunction (49). In a prior study regarding lung metastasis formation in breast cancer, it was shown that sensory vagal activity significantly diminishes both SP levels and the metastatic burden (8). A later study showed that drug-induced vagal activity also decreases the metastatic burden and metastasis formation in the 4THM breast cancer model (9). Therefore, it has been proposed, functional capsaicin-sensitive afferents constitute a gatekeeper mechanism which limits metastatic propagation in breast cancer *via* local neuropeptide release (50). Furthermore, it was found that capsaicin-induced desensitization results in a less favorable gene expression profile in breast cancer, potentially due to the diminished activation of certain genes pivoting towards inhibition of cancer growth (50–52). However, it has been shown that tumor cells also express SP, and SP levels are elevated in breast cancer due to the lacking inhibitory effect of the RE1-silencing transcription factor (REST) in SP-expression (53). Since REST is downregulated in breast cancer, this results in an increased intratumoral SP-level. Considering the antitumoral effects of SP at lower levels, it has been proposed that intact SP stimulates tumorigenesis, while its bioactive fragments resulting from enzymatic hydrolysis can suppress tumor growth (54, 55). While capsaicin-sensitive afferents are a pivotal source of CGRP, expression of this vasoactive neuropeptide was also not significantly lower in desensitized animals. However, it has been shown, breast cancer cells themselves strongly express CGRP (56). Thus, in conclusion, this non-neural source of the peptide maintains its concentration within the tumor tissue. In a similar manner, while somatostatin is released by sensory nerve endings in significant quantities, the gut and central nervous system are considered to be their primary source in rodents (57). Comprehensively, our findings indicate that intratumoral neuropeptide levels are primarily influenced by non-neural sources. Further studies using both neuropeptide gene-deficient mice and tumor cell lines are warranted to differentiate how tumoral and systemic, yet non-neural neuropeptide expression, contributes to their production and biologic action in breast cancer.

Capsaicin sensitive-sensory nerves influence tumor growth, and vascular permeability in this orthotopic model of 4T1 breast cancer. The increased vascular leakage observed within the tumors of desensitized mice is likely due to the imbalance of local neurogenic vasoregulation, which in turn, can facilitate tumor vascular bed disruption. On the other hand, intratumoral protease expression was also not significantly different in our experiment, accompanied by a maintained level of neuropeptides in the tumor tissue. Finally, as demonstrated by the histopathological analysis long-term tumor growth, cellular profile and viability were not significantly influenced by the desensitization of these nerve terminals.

Admittedly, our study has several limitations. Most importantly, only the levels of selected key neuropeptides were measured, which was dictated by the limited amount of tumor tissue available for ex vivo radioimmunoassay. Furthermore, the molecular underpinnings of the observed increased intratumoral vascular leakage in RTX-pretreated mice remain unclear. Additionally, the MRI protocol used is ill-suited for measuring the peritumoral edema, which may explain the diverging results compared to the tumor size measurements obtained through the use of a caliper.

In summary, capsaicin-sensitive sensory nerves exert important and beneficial vasoregulatory effects on the tumor tissue, which reduces pathological vascular hyperpermeability and thereby early phase tumor growth (and potentially peritumoral edema). However, systemic desensitization in itself has no direct effect on tumor structure and cellular viability. These findings, along with prior evidence underline capsaicin-sensitive afferents and functioning sensory innervation of the tumor microenvironment possess homeostatic and antitumoral properties. Therefore, caution must be maintained while pharmacologically targeting these nerve terminals (e.g., use of TRPV1 antagonist analgesic candidates), in order to avoid potential detrimental effects in facilitating tumorigenesis. On a separate note, the increased intratumoral leakage can also significantly influence effective drug delivery into the tissue and can facilitate selective accumulation of large molecule therapeutics within the tumor.

## Data Availability Statement

The datasets presented in this article are available from the corresponding author on reasonable request.

## Ethics Statement

The animal study was reviewed and approved by Animal Welfare Committee of the University of Pécs and the National Scientific Ethical Committee on Animal Experimentation of Hungary. Licensed by the Government Office of Baranya County (license No. BA02/2000-32/2018).

## Author Contributions

Study design: BB, ZH, PH, and NB. Preparation and inoculation of tumor cells: NB, LD, and ÉS. Data acquisition: NB, BB, CS, PB, and GK. Data analysis: BB, NB, ZH, PH, and CS. Manuscript writing: BB, NB, PH, ZH, and ÁS. All authors contributed to the article and approved the submitted version.

## Funding

This work was supported by grants EFOP-3.6.2-16-2017-00008, “The role of neuro-inflammation in neurodegeneration: from molecules to clinics”, EFOP-3.6.1-16-2016-00004, “Stay Alive” GINOP-2.3.2.-15-2016-00048, “PEPSYS” GINOP-2.3.2-15-2016-00050, Hungarian Brain Research Program 2017-1.2.1-NKP-2017-00002, and University of Pécs, Medical School grant KA-2015-20. BB was supported by the János Bolyai Research Scholarship of The Hungarian Academy of Sciences and the ÚNKP-20-5-PTE-540 New National Excellence Program of the Ministry for Innovation and Technology. GK was supported by the ÚNKP-20-3-II-PTE-734 New National Excellence Program of the Ministry for Innovation And Technology from the source of the National Research, Development and Innovation Fund. This work was supported by the university collaboration program of the Lorand Eotvos Research Network.

## Conflict of Interest

The authors declare the research was conducted in the absence of any commercial or financial relationships which may be considered as a potential conflict of interest.
